# Impact of epidural analgesia on outcomes of vaginal birth after cesarean delivery

**DOI:** 10.1007/s00404-025-07959-y

**Published:** 2025-01-30

**Authors:** Tamar Eshkoli, Merav Jacobs, Alla Saban, Yael Baumfeld, Renana Ben Shushan–Amor, Zehava Yohay, Adi Y. Weintraub

**Affiliations:** 1 Faculty of Health Sciences, Ben-Gurion University of the Negev, Beer-Sheva, Israel; 2 Department of Obstetrics and Gynecology, Soroka University Medical Center, Beer-Sheva, Israel; 3 Medical School for International Health, Ben-Gurion University of the Negev, Beer-Sheva, Israel

**Keywords:** Epidural analgesia (EA), TOLAC, Uterine rupture, Vaginal delivery, VBAC

## Abstract

**Primary objective:**

To assess the association between Epidural Analgesia (EA) during Vaginal Birth After Cesarean (VBAC) and delivery mode (spontaneous or instrumental vaginal delivery). Secondary objectives include maternal and neonatal outcomes.

**Methods:**

In this retrospective population-based cohort study, all women who underwent a VBAC with and without EA, between the years 1996–2016 at the Soroka University Medical Center (SUMC) were included. Women who delivered by cesarean section (elective and non-elective) and those who gave birth to a newborn with chromosomal abnormalities or major malformations, and multifetal gestations were excluded. Demographical, clinical, and obstetrical characteristics were recorded, and pregnancy complications and adverse perinatal outcomes were compared between the groups. The primary outcome was the type of delivery. Univariate analysis was followed by a multivariate analysis to control for confounders. A *p* value of < 0.05 was considered statistically significant.

**Results:**

During the study period, 17,516 women who have had a previous CS met the inclusion criteria, of which 15% (*n* = 2652) used EA during labor, while the rest of the cohort 85% (*n* = 14,864) were non-EA users. Women in the EA group had higher rates of instrumental delivery and postpartum hemorrhage (PPH) as well as higher rates of oxytocin augmentation and a longer second stage of labor. The use of EA was found to be an independent risk factor for instrumental delivery after controlling for maternal age, ethnicity, parity, DM (diabetes mellitus), hypertensive disorders of pregnancy, oxytocin augmentation, prolonged second stage of labor, induction of labor, fertility treatments and oligohydramnios. However, no significant differences were observed regarding neonatal outcomes including perinatal mortality, birth weight, Apgar scores and shoulder dystocia rates.

**Conclusion:**

EA for women undergoing a VBAC was associated with higher rates of instrumental delivery and PPH, oxytocin augmentation and a longer second stage of labor compared with women without EA. However, neonatal outcomes did not differ between the groups.

## What does this study add to the clinical work

**Table:** 

EA for women undergoing a VBAC was associated with a significant increase in adverse pregnancy outcomes compared to women who didn't receive an EA.	

## Introduction

The incidence of cesarean section delivery (CS) has been increasing worldwide over the past few decades [1]. CS is the most common surgery performed in the United States, with rates rising from 5% in 1970 to 31.9% in 2016 [2]. Repeat CS is the most significant factor contributing to the rise in CS rates [3].

Multiple CS are associated with increased risk, including injury to bladder or bowel, hemorrhage, uterine rupture, and hysterectomy [4, 5]. These adverse outcomes may have a significant effect on the course of future pregnancies [6]. Additionally, elective repeat CS is linked to thromboembolic events and placenta accreta [7].

Overall rates of maternal harms were low for both trial of labor and elective repeat cesarean delivery, with a slightly increased risk of uterine rupture associated with the trial of labor [8]. Failed trials of labor after CS (TOLAC) increase the risk of postpartum infection, uterine atony, and wound separation compared to planned repeat CS [9].

Epidural analgesia (EA) is considered the gold standard for labor pain management. Benefits of EA include reduced maternal exhaustion, effective pain relief throughout labor, decreased maternal anxiety and fear of childbirth, and a rapid anesthesia option if CS becomes necessary [10, 11]. Thus, it is common to use EA during VBAC especially in deliveries with a higher risk of being protracted or dysfunctional [12]. Furthermore, EA is possibly a significant adjunct to a successful VBAC [13]. According to the recent literature, EA is considered to be safe for both the mother and neonate and does not mask signs or delay diagnosis of uterine rupture as previously thought [14, 15].

However, EA may have adverse effects on the course of labor even in vaginal deliveries that are not TOLAC. These changes, such as reduced pelvic muscle tone, hormonal changes, and intrapartum maternal fever [16] may contribute to dysfunctional and prolonged labor [17]. Other studies have shown the association between EA and an increased number of instrumental deliveries [18].

In this study, we aim to investigate whether there is an association between EA and adverse pregnancy outcomes among women who underwent a VBAC.

## Materials and methods

### Study design

A retrospective cohort study was conducted at Soroka University Medical Center (SUMC), examining all women who underwent vaginal birth after cesarean (VBAC) between 1996 and 2016, comparing those who received epidural analgesia (EA) with those who did not. SUMC is a tertiary hospital located in the Negev (southern district of Israel), which constitutes 65.5% of the land of Israel. As the only tertiary teaching hospital in the Negev, SUMC serves the entire population of the region (14.4% of Israel’s population) [19]. As a result, the study is based on nonselective population-based data. The institutional review board has approved the study (in accordance with the Helsinki Declaration 1975, revision 2013, # 0275-21-SOR, date of approval—6.1.12022).

### Study population

The study group was defined as women who underwent a previous single CS who are older than 18 years and delivered between 37 and 42 weeks of gestation by a spontaneous or instrumental vaginal delivery. Women who delivered by CS (elective and non-elective), gave birth to a neonate with chromosomal abnormalities or major malformations, pregnancies with multifetal gestation and women who had a lack of prenatal care (LOPC) were excluded from the study. Lack of prenatal care was defined as the absence of any prenatal tests throughout the pregnancy.

### Outcomes

Outcomes of interest primarily included the type of delivery: instrumental vaginal delivery (vacuum-assisted delivery) or spontaneous vaginal delivery. Secondary outcomes included maternal and neonatal morbidities such as: uterine rupture, early postpartum hemorrhage (PPH) (defined as blood loss more than 500ml during vaginal delivery and more than 1000ml during CS, or use of uterotonics(, need for blood transfusion, perineal lacerations (specifically 3rd and 4th degree lacerations) low Apgar scores (< 7) at one and five minutes and neonatal death.

### Data collection

Data was collected from the SUMC computerized perinatal database of the Department of Obstetrics and Gynecology. Patient demographics, as well as clinical and obstetrical data including delivery and perinatal outcomes were entered into the database by the attending obstetricians immediately after delivery, to ensure maximum accuracy. Pregnancy complications including large for gestational age neonate (LGA), suspected macrosomia (estimated fetal weight > 4000 g), Preeclampsia/gestational hypertension), stillbirth, instrumental delivery, perineal tears and shoulder dystocia were evaluated. All diagnoses were classified according to the International Classification of Diseases (ICD-9).

During the study period, vacuum-assisted deliveries were the only type of instrumental delivery performed.

The second stage of labor was defined as prolonged if it lasted more than 3 h for patients with no previous vaginal delivery who delivered under epidural anesthesia, and as prolonged if it lasted more than 2 h for patients with a history of normal vaginal delivery (before or after cesarean section) and without epidural anesthesia, according to the definitions that were accepted during the study period.

Women who had multiple deliveries during the study period may have been included in the analysis more than once, as each delivery was considered a separate event.

### Statistical analysis

Data were analyzed using the SPSS package 23rd edition. The initial analysis was performed by using descriptive statistics, followed by advanced analytical statistics. Unadjusted rates and means were compared using Pearson Chi-square, Student’s *t* test, and Mann–Whitney *U* test, as appropriate for categorial normally distributed continuous variables and continuous variables without normal distribution, respectively. Variables found to be significant in the univariate analysis, in addition to variables with clinical significance, were included in the multivariable logistic regression analysis. Adjusted odds ratios (OR) and 95% confidence intervals (CI) were computed. *p* values < 0.05 were considered statistically significant.

## Results

During the study period, 17,516 women who have had a previous CS met the inclusion criteria, of which 2652 (15%) used EA during labor (study group), while the reminder of the cohort 14,864 (85%) were non-EA users (comparison group). Table 1 presents the demographical and clinical characteristics of the study population. Women in the study group were slightly older (*p* < 0.001) with lower parity (*p* < 0.001) and were more likely to conceive after fertility treatments (*p* < 0.001). Most were of Jewish ethnicity (77.6%) and only 22.4% were of Bedouin Arab ethnicity (*p* < 0.001).

**Table 1:** Demographic and clinical characteristics in post CS women with and without epidural analgesia

Characteristics		Epidural analgesia *n* = 2652	No epidural analgesia *n* = 14,864	*p* value
Maternal age (years, mean ± SD)		30.38 ± 4.88	29.96 ± 5.45	< 0.001
Jew *n* (%)		2057 (77.6)	4615 (31.0)	< 0.001
Bedouin *n* (%)		595 (22.4)	10,249 (69.0)	
Median parity		2.99 ± 1.56	4.82 ± 2.58	< 0.001
Gestational age (weeks)		39.27 ± 1.58	39.22 ± 1.88	0.23
Fertility tx type *n* (%)	OI	13 (0.5)	56 (0.40)	< 0.001
	IVF	68 (2.6)	70 (0.5)	< 0.001
DM % *n* (%)		21 (0.8)	202 (1.4)	< 0.001
Years *n* (%)	1988–2000	536 (19.9)	6202 (40.2)	
	2001–2005	437 (16.2)	2987 (19.4)	
	2006–2010	659 (24.4)	2661 (17.3)	
	2011–2016	1068 (39.6)	3574 (23.2)	< 0.001

*DM diabetes mellitus, *IVF* in vitro fertilization, *OI* Ovulation induction

Table 2 outlines the obstetrical and labor characteristics of the study population. No significant differences were observed between the groups regarding hypertensive disorders, maternal diabetes and intrauterine growth restriction (IUGR) (defines as suspected fetal weight below 3th percentile).

**Table 2:** Obstetrical and labor characteristics of the study population

		Epidural analgesia *n* = 2652	No epidural analgesia *n* = 14,864	*p* value
Hypertensive disorders in pregnancy (pre-eclampsia, gestational HTN, chronic HTN) *n* (%)		99 (3.7)	638 (4.3)	0.19
PET *n* (%)	All	89 (3.3)	459 (3.0)	0.37
	Eclampsia%	0 (0.0)	1 (0.0)	1.00
	Mild %	79 (2.9)	372 (2.4)	0.12
	Severe %	10 (0.4)	86 (0.6)	0.22
GDM *n* (%)		117 (4.4)	678 (4.6)	0.73
Polyhydramnios (AFI > 24) *n* (%)		55 (2.1)	408 (2.7)	0.05
Oligohydramnios (AFI < 5) *n* (%)		66 (2.5)	250 (1.7)	0.01
IUGR *n* (%)		42 (1.6)	174 (1.2)	0.08
Macrosomia *n* (%)		103 (3.9)	615 (4.1)	0.54
Placental abruption *n* (%)		1 (0.0)	36 (0.2)	0.04

*AFI amniotic fluid index, *PET* Preeclampsia/gestational hypertension, *GDM* gestational diabetes mellitus, *IUGR* intra-uterine growth restriction

Table 3 describes pregnancy and neonatal outcomes in the study population. Higher rates of instrumental delivery were noted among the study group (11.3% vs. 4.7%; *p* < 0.001). Differences in labor characteristics were also found; the study group had higher rates of labor induction (16.2% vs.10.3%, *p* < 0.001), oxytocin use during labor (4.3% vs. 3.4%; *p* < 0.001) and longer mean duration of the second stage of labor (2.1% vs 1.2%; *p* < 0.001) compared to the non EA group. Furthermore, women in the study group had higher rates of PPH (0.8% vs. 0.4%; *p* = 0.01) and overall perineal tears (30.6% vs. 18.5%; *p* value < 0.001) including 3rd and 4th degree tears (0.3% vs. 0.1%; *p* value < 0.001). There was no difference regarding neonatal outcomes between the groups except for a slightly increased mean birth weight in the study group (3257.57 ± 459.87 vs. 3210 ± 497.10; *p* < 0.001).

**Table 3:** Pregnancy and neonatal outcomes in post CS women with and without epidural analgesia

		Epidural analgesia *n* = 2652	No epidural analgesia *n* = 14,864	*p* value
Mode of delivery	Spontaneous vaginal deliveries *n* (%)	2352 (88.7)	14,164 (95.3)	< 0.001
	Instrumental vaginal delivery *n* (%)	300 (11.3)	700 (4.7)	< 0.001
Induction of labor *n* (%)		428(16.2)	1535(10.3)	< 0.001
Oxytocin use during labor *n* (%)		114 (4.3)	499 (3.4)	0.02
NPL1 *n* (%)		7 (0.3)	4 (0.0)	< 0.001
NPL2 *n* (%)		57 (2.1)	175 (1.2)	< 0.001
NRFHR *n* (%)		24 (0.9)	161 (1.1)	0.41
PPH *n* (%)		20 (0.8)	57 (0.4)	0.01
3 and 4 degree perineal tears *n* (%)		9 (0.3)	16 (0.1)	0.01
All tears *n* (%)		812 (30.6)	2748 (18.5)	< 0.001
Perinatal mortality *n* (%)		22 (0.8)	143 (1.0)	0.52
LGA *n* (%)		96 (3.6)	554 (3.7)	0.32
Birth weight (grams, mean ± SD)		3257.57 ± 459.87	3210 ± 497.10	< 0.001
Low Apgar score (< 7) *n* (%)		9 (0.3)	44 (0.3)	0.74
At 1 min		8.84 ± 0.77	8.86 ± 0.71	0.15
At 5 min		9.94 ± 0.45	9.94 ± 0.44	0.69
Shoulder dystocia *n* (%)		1 (0.0)	33 (0.2)	0.05

*PPH post partum hemorrhage, *LGA* large for gestational age, *NPL* non-progressive labor, *NRFHR* non-reassuring fetal heart rate

Table 4 presents the results of a multivariate analysis, with instrumental deliveries as the outcome variable. This analysis adjusts for the background characteristics that are considered to be significant mediators for the mode of delivery. EA use was independently associated with higher rates of instrumental deliveries (aOR = 2.65, [95%CI 2.22–3.16]; *p* < 0.001), after controlling for maternal age, ethnicity, parity, DM, hypertensive disorders of pregnancy, oxytocin use, prolonged second stage of labor, induction of labor, fertility treatments, and oligohydramnios.

**Table 4:** Instrumental vs. spontaneous vaginal mode of delivery in the presence of epidural analgesia: multivariate analysis

	aOR	95% CI	*p* value
Epidural analgesia	2.65	2.22–3.16	< 0.001
Maternal age	1.05	1.04–1.07	< 0.001
Jewish ethnicity	0.34	0.28–0.41	< 0.001
DM	1.20	0.88–1.65	0.25
Hypertensive disorders of pregnancy	0.75	0.50–1.13	0.16
Oxytocin	0.75	0.55–1.03	0.08
NPL2%	369.75	216.64–631.10	< 0.001
Parity	0.72	0.69–0.75	< 0.001
Induction of labor	1.06	0.65–1.75	0.81
Fertility tx	0.66	0.35–1.24	0.20
Oligohydramnions	2.65	2.22–3.16	< 0.001

*DM diabetes mellitus, *NPL* non-progressive labor

## Discussion

Our study included 17,516 women who underwent a VBAC delivery. Of these participants, 15% used EA during labor. No significant differences were found between the groups regarding pregnancy complications or neonatal outcomes. However, in the study group there were higher rates of instrumental deliveries and PPH as well as higher rates of oxytocin use and a longer second stage of labor. After controlling for potential confounders, EA use was independently associated with higher rates of instrumental deliveries. In general, the use of EA is considered safe in women who desire to undergo a TOLAC and has been proven in previous studies to be an important contributor for achieving a VBAC and avoiding a recurrent CS [13]. However, our study has shown that among women who achieved a VBAC, those who did not use EA had a shorter duration of labor and a lower likelihood of instrumental delivery. These findings are compatible with previous studies that showed an increased rate of instrumental delivery ranging between 10 and 56% [13, 20–23]. In one study by Grisaru-Granovsky, this increase was statistically significant, with the odds of instrumental delivery being three times higher in the EA group compared to the non-EA group [13].

EA increases the risk of instrumental delivery for both women attempting VBAC and those with previous vaginal deliveries [18, 20–22]. There are several possible pathophysiological explanations; First, the use of EA can lead to relaxation of the pelvic muscles, thus it may be more difficult for the fetus to descend through the birth canal [23]. In addition, the use of EA can prolong the second stage of labor, as was also demonstrated in our study, and this prolongation has been associated with an increased risk for instrumental intervention [24, 25]. Furthermore, EA can lead to an increase in the use of oxytocin during labor, a change that was also observed in our study. This can result in uterine hyper-stimulation, which may compromise uteroplacental blood flow and oxygen supply to the fetus. As a result, fetal distress may occur, increasing the likelihood of requiring instrumental assistance, such as vacuum extraction, to facilitate delivery [26]. Finally, EA may also impair a woman's ability to push effectively during the second stage of labor which can lead to the need for instrumental intervention to advance the labor [27]. It is important to note that a previous CS may increase the likelihood of instrumental intervention independently due to post-operative anatomical changes such as adhesions or scar tissue, which can affect the shape and position of the uterus, making vaginal delivery more difficult [28]. In addition, a previous CS can impact fetal position and size, making the vaginal delivery more challenging [29].

According to these mechanisms, other maternal outcomes besides instrumental delivery were associated with EA among women having a VBAC. The outcomes in our study include increased use of oxytocin to augment labor, longer second stage of labor and increased rates of perineal tears, including severe 3rd and 4th degree tears and increased rates of PPH. These associations were observed in previous studies as well [30]. In contrast, no significant associations between EA and adverse neonatal outcomes were demonstrated in our study, which is in accordance with previous literature [13]. The slight difference in birth weight observed between the groups is likely due to other factors and not directly related to epidural use. However, previous studies have shown that the use of EA during a TOLAC is associated with lower rates of recurrent CS and a successful VBAC [13]. Our study did not include patients undergoing TOLAC that resulted in a CS. However, according to previous literature, patients should be informed that the use of EA during TOLAC can help prevent a recurrent CS, but at the same time, may increase the risk for instrumental delivery, the need for augmentation with oxytocin, prolonged labor, and increased rate of PPH and perineal tears.

Hence, the decision if to use EA among women undergoing TOLAC should be individualized and based on a comprehensive evaluation of the woman's overall health, the specific circumstances of her pregnancy and delivery, woman's pain tolerance and her personal preferences [31, 32],

### Limitations and strengths

Due to the retrospective nature of the study, which relied on a computerized database, we were unable to distinguish between elective and emergent CS. While the use of a computerized database reduces the risk of errors, it does not eliminate the potential for information bias entirely. This limitation, coupled with the inability to differentiate between types of cesarean sections, narrowed our ability to assess the effect of EA on achieving a successful TOLAC. Moreover, our study population consisted only of women who had vaginal deliveries, either through spontaneous labor or vacuum-assisted delivery. Cases which ended up in cesarean section were excluded from both the study and the control groups. As a result, we focused solely on TOLAC which resulted in VBAC. This limitation also restricted our ability to evaluate the broader impact of EA on successful TOLAC outcomes. However, it allowed us to concentrate on VBAC-specific outcomes, ensuring a more precise evaluation within the scope of our research. Future studies should aim to investigate the effect of EA on achieving a successful TOLAC, not necessarily limited to VBAC, while also addressing potential sources of information bias.

Another limitation is the extended time period that was included in the database. Trends based on professional guidelines and local institutional protocols may have changed over time and may have impacted the results, as demonstrated in Table 1. However, we believe that if such changes have occurred, they would have equally influences patients from both the study group and the comparison group.

Another limitation of this study is that some women may have been included in the analysis multiple times if they had more than one delivery during the study period. This could potentially introduce bias.

On the other hand, there are several noteworthy strengths to our study. The data we used were retrieved from a computerized database, incorporating a considerable patient pool from a single tertiary medical center. This substantially diminishes the likelihood of erroneous outcome data. Secondly, we have been able to merge maternal and neonatal data in our dataset, giving us the ability to regulate for various pregnancy and delivery variables. Additionally, the population in our study uses epidural anesthesia less frequently due to various characteristics of the target population, including lower access to healthcare services and a cultural background where the use of epidural anesthesia is less accepted, compared to the rates reported in the existing literature [33, 34].

More studies are needed to investigate the impact of EA on women who have had a previous CS delivery and choose to follow with a vaginal delivery in their subsequent birth. The gap in literature currently lies in understanding the long-term maternal and neonatal effects. These studies should focus on the following maternal long-term outcomes; maternal satisfaction with pain management, maternal morbidity (e.g. urinary and/or fecal incontinence, pelvic organ prolapse), the course of future pregnancies (such as the risk for another CS or the likelihood for a successful vaginal delivery) and maternal mental health and well-being, including depression, anxiety, and stress levels. Moreover, fetal long-term outcomes should include neonatal development, including the risk of developmental delays or behavioral issues and mother-infant bonding processes.

## Conclusion

The use of EA during TOLAC has been associated with increased rates of instrumental delivery, third- and fourth-degree perineal tears, PPH, as well as higher utilization of oxytocin and prolonged duration of the second stage of labor. However, counseling regarding EA should not be limited to women who successfully achieve a VBAC. It is essential to consider all possible outcomes of TOLAC, including cesarean delivery, when discussing the use of EA. Additionally, baseline characteristics should be considered in future analyses to determine whether observed outcomes, including PPH and third- and fourth-degree perineal tears, are significantly different when adjusting for these variables. Further research is needed to explore the long-term maternal and neonatal effects of EA across all TOLAC outcomes, including those who ultimately require cesarean delivery.

## Data Availability

No datasets were generated or analysed during the current study.
